# In-vitro and in-vivo imaging of coronary artery stents with Heartbeat OCT

**DOI:** 10.1007/s10554-020-01796-7

**Published:** 2020-02-28

**Authors:** Leonardo Cecchetti, Tianshi Wang, Ayla Hoogendoorn, Karen T. Witberg, Jurgen M. R. Ligthart, Joost Daemen, Heleen M. M. van Beusekom, Tom Pfeiffer, Robert A. Huber, Jolanda J. Wentzel, Antonius F. W. van der Steen, Gijs van Soest

**Affiliations:** 1 Biomedical Engineering, Thoraxcenter, Erasmus MC, P.O. Box 2040, 3000 CA Rotterdam, The Netherlands; 2 Department of Cardiology, Thoraxcenter, Erasmus MC, Rotterdam, The Netherlands; 3 Optores GmbH, München, Germany; 4 Institut für Biomedizinische Optik, Universität Zu Lübeck, Lübeck, Germany; 5 Shenzhen Institutes of Advanced Technology, Chinese Academy of Sciences, Shenzhen, China; 6 Department of Imaging Science and Technology, Delft University of Technology, Delft, The Netherlands

**Keywords:** Drug-eluting stent, Optical coherence tomography, Innovation

## Abstract

To quantify the impact of cardiac motion on stent length measurements with Optical Coherence Tomography (OCT) and to demonstrate in vivo OCT imaging of implanted stents, without motion artefacts. The study consists of: clinical data evaluation, simulations and in vivo tests. A comparison between OCT-measured and nominal stent lengths in 101 clinically acquired pullbacks was carried out, followed by a simulation of the effect of cardiac motion on stent length measurements, experimentally and computationally. Both a commercial system and a custom OCT, capable of completing a pullback between two consecutive ventricular contractions, were employed. A 13 mm long stent was implanted in the left anterior descending branch of two atherosclerotic swine and imaged with both OCT systems. The analysis of the clinical OCT images yielded an average difference of 1.1 ± 1.6 mm, with a maximum difference of 7.8 mm and the simulations replicated the statistics observed in clinical data. Imaging with the custom OCT, yielded an RMS error of 0.14 mm at 60 BPM with the start of the acquisition synchronized to the cardiac cycle. In vivo imaging with conventional OCT yielded a deviation of 1.2 mm, relative to the length measured on ex-vivo micro-CT, while the length measured in the pullback acquired by the custom OCT differed by 0.20 mm. We demonstrated motion artefact-free OCT-imaging of implanted stents, using ECG triggering and a rapid pullback.

## Introduction

Percutaneous coronary intervention is the standard procedure for the treatment of acute coronary syndrome. The procedure is carried out under X-ray angiography guidance to visualize the coronary artery lumina. Yet, X-ray angiography has some limitations: it provides 2D projections of a 3D structure, it gives no information about the vessel wall geometry and composition, and it has a limited resolution [1]. These limitations can be overcome with intravascular imaging: intravascular ultrasound (IVUS) [2] and optical coherence tomography [3–7] (OCT) are commercially available modalities.

These technologies provide detailed imaging of the inside of the coronary vasculature, which has led to improved clinical outcomes in terms of major adverse cardiac events and even mortality [8], immediately and in the long term [9–13], and of post-procedure fractional flow reserve (FFR) [14].

Among the imaging technologies available, OCT offers, with an axial resolution of 20 µm, the highest amount of detail to visualize the vessel wall morphology. However, motion artefacts generated by ventricular contractions during imaging and undersampling in the pullback direction can affect the OCT volumetric renderings [15]. These artefacts occur as the pullback speed and frame rate dictate that a pullback acquisition is performed over 2–3 s, during which the heart beats a few times. Longitudinal and transverse motion of the catheter relative to the vessel under study may generate deformations in the imaged volume [16] that can affect local measurements of for instance plaque length, which may affect stent selection. Undersampling results from the frame pitch being greater than the lateral image resolution, and leads to a disconnected appearance of microscopic features along the pullback [17]. A previous realization of high-speed OCT stent imaging demonstrated artefacts in the rendered stent originating in cardiac motion, but could not address undersampling due to limitations in frame rate [18].

In this study, we quantified the effects of motion artefacts on OCT image quality and assessed whether ECG triggering, together with high acquisition speed, mitigated these effects. The study consists of three parts: we assessed the effect of cardiac motion by comparing the longitudinal geometric measure of implanted coronary stents with their nominal lengths [19] in clinical data. We then replicated the observed length mismatches in a cardiac motion simulation, evaluating the effect of ECG triggering and very fast pullbacks, compared to conventional acquisitions, in a benchtop experiment. Finally, we compared the lengths of stents implanted in a large atherosclerotic animal model in vivo with the two versions of OCT imaging.

## Methods

### Quantification of OCT-measured length mismatch in clinical data

The influence of motion artefacts in OCT images of implanted coronary stents was assessed by comparing the lengths of implanted stents as measured by OCT with their nominal lengths. OCT data were gathered from the clinical database at Erasmus MC. A total of 101 pullbacks (101 patients; 61 LAD, 16 LCX, 23 RCA and 1 diagonal branch), acquired with a commercial OCT system (Ilumien™ Optis™, Abbott, Abbott Park, Illinois, USA) during PCI directly after implantation of a single stent, were included. All data sets were acquired with calibrated Z-offsets and had good flush quality. Measurements were made by a trained observer, who was blinded to the nominal lengths. The stent lengths were calculated by counting the total number of frames that contained at least one stent strut, consistent with clinical practice, and multiplying by the frame pitch as specified by the manufacturer. Subsequently, the OCT-measured lengths were compared with the nominal measures.

The data were processed using regression analysis to study the relation between OCT-measured stent lengths and nominal lengths.

### Preclinical OCT imaging

**Fig. 1:**
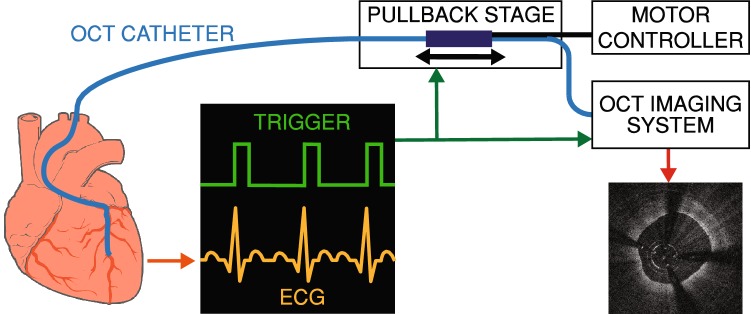
Heartbeat OCT schematic: image acquisition is triggered by the ECG signal, completing the scan of the coronary artery between two subsequent heart contractions

We implemented an OCT system, which we have called Heartbeat OCT (Fig. 1), that acquires data approximately 15 times faster than conventional OCT (A-scan rate of 1.55 MHz, compared to about 100 kHz in commercial systems) and 6 times than other prototypes [18], which enables imaging at 3000 frames per second (fps) in a pullback at 100 mm/s, compared to 180 fps and 36 mm/s of commercial systems. The Heartbeat OCT was previously described in detail [16, 20]. It relies on three key elements: (1) a Fourier Domain Mode Locked (FDML) laser [21], (2) a distally actuated imaging catheter [22] and (3) ECG triggering of the scan. This scan sequence enables imaging of a relevant section of the coronary artery during the diastolic phase of the cardiac cycle, when there is no rapid tissue motion due to ventricular contraction.

For reference, a conventional OCT system was used.

### Cardiac motion simulation

#### Numerical simulation

**Fig. 2:**
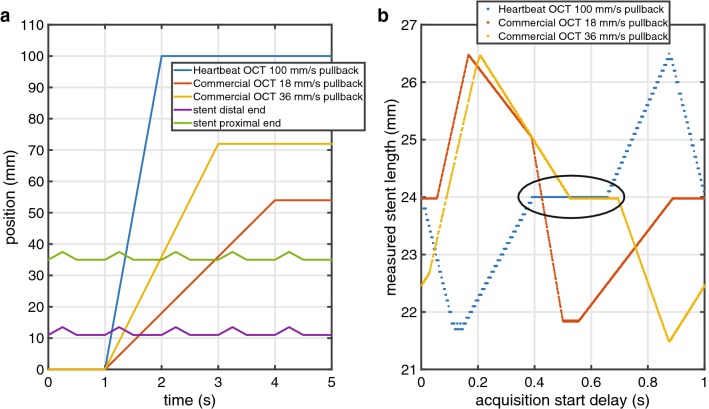
Cardiac motion numerical simulation. **a** Simulation outline: the lines depict the position in time of stent edges and OCT catheters tip in time. **b** Simulation result: measured stent length values obtained at different pullback speeds with random trigger starts (true value 24 mm, 60 BPM, tissue velocity 10 mm/s). The black oval indicates the range of acquisition delays where Heartbeat OCT completes the pullback within the resting phase

The variable position of a stented vessel due to cardiac motion was numerically simulated as a one-dimensional oscillatory linear motion pattern, implemented in MATLAB R2018a (The Mathworks, Natick, MA, USA). The position of the catheter tip was described as linear motion in the same direction. Stent lengths were computed in this simulation by finding the first intersection point between the oscillatory line describing the distal stent edge position and the straight line of the catheter tip position, and similarly, between the last intersection point of the proximal stent edge position and catheter tip (Fig. 2a). The distance between these two intersection points was taken to be the simulated stent length. Asynchronous acquisition was studied by varying the time offset of the catheter tip trajectory relative to the cardiac cycle in 1 ms increments, accumulating the results and computing descriptive statistics. Heart rates between 50 and 100 beats per minute and tissue velocities from 10to 40 mm/s were simulated. Motion parameters (speed, frequency, amplitude, rest time) were chosen to simulate human cardiac motion, modelled on tissue velocities measured in M-mode echocardiography and angiography [19, 23].

#### In vitro simulation

**Fig. 3:**
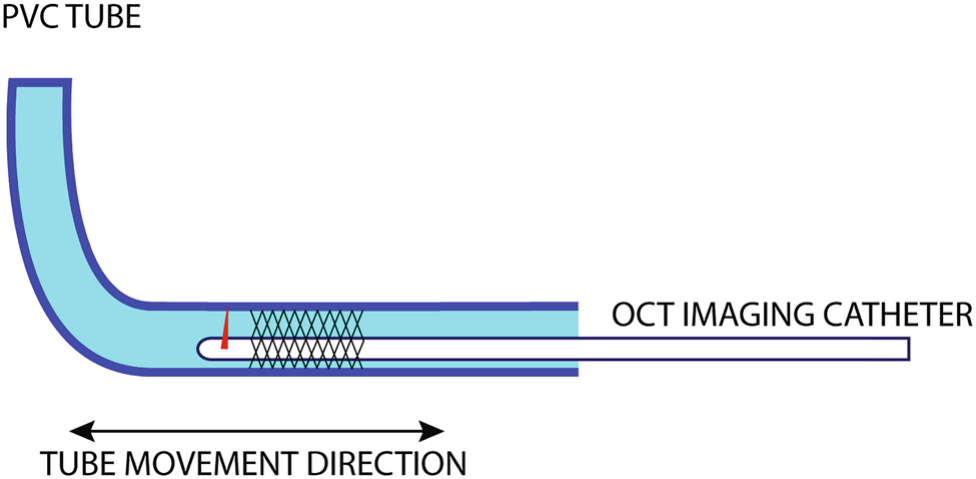
Bench-top experiment schematic: a coronary stent in deployed in a PVC tube; the tube translates longitudinally during the OCT acquisition, simulating relative motion between imaging catheter and the imaged vessel

Reproducible, controlled cardiac motion was simulated by mounting a vessel-mimicking tube (inner diameter 3.0 mm, PVC) on a linear motor stage (EACM6D10AZAC, Oriental Motor Co. Ltd., Tokyo, Japan). A 5.0 × 26 mm coronary stent (Resolute Onyx™, Medtronic, Minneapolis, Minnesota, USA) was deployed inside the tube, which was then filled with water, in order to acquire the OCT images in a medium with a refractive index very close to that of saline solution. The linear motor stage was programmed to simulate cardiac motion of a vessel relative to the imaging catheter by translating the tube along its longitudinal axis (Fig. 3).

The length of the stent was measured with a calliper and by performing repeated pullbacks (N = 10) without motion with a commercial OCT system. In the OCT images, the length of the stent was measured by counting the number of frames that contained at least one stent strut and averaging the results of all pullbacks. The pullback speed was 18 mm/s, frame pitch 100 µm.

**Fig. 4:**
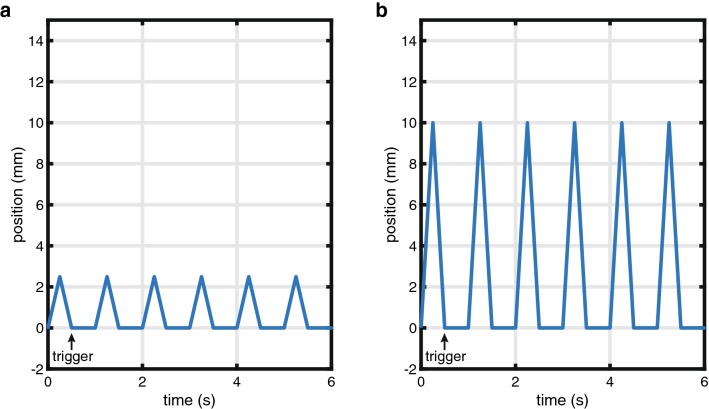
Heartbeat OCT triggering in the bench-top experiment, tissue speed. **a** 10 mm/s, **b** 40 mm/s

A selection of motion parameter sets that were simulated in Matlab were programmed in this setup. For every selection of parameters, four sets of OCT scans were performed with both the commercial OCT system (pullback speeds 18 and 36 mm/s, frame pitch 100 and 200 µm) and with the Heartbeat OCT system (pullback speed 100 mm/s, frame pitch 33 µm), with and without synchronisation of the start of the pullback (Fig. 4) to the cardiac cycle. For every selection of motion parameters, 20 pullbacks were recorded.

#### Data analysis

To quantify the distortion introduced by the movement of the tube, the length of the deployed stent was measured in every pullback. We computed, for each set of pullbacks, average length, standard deviation and root mean square deviation from the real value. Length errors are reported as (signed) mean ± standard deviation.

### In vivo imaging of stents

The in vivo preclinical experiments were approved by the Erasmus MC animal ethics committee (DEC-EMC3125(109–12-25)) and the animals were treated according to National Institutes of Health Guide for the care and use of laboratory animals [24]. The animals were adult (3 years old) Familial-hypercholesterolemia Bretonchelles Meishan (FBM) pigs [25] (n = 2, castrated males).The pigs were fed with a high-fat diet for the 9 months preceding the experiments.

Twenty-eight days before the imaging experiment, stents (3.0 × 13 mm, Orsiro, Biotronik, Lake Oswego, Oregon, USA) were implanted in the left anterior descending (LAD) at a location with early-stage plaque. All animals were treated with acetylsalicylic acid 250 mg and clopidogrel 300 mg (Plavix, Sanofi-Aventis, Paris, France) during follow up and 75 mg clopidogrel daily until the imaging experiment.

After an overnight fast, sedation was done with xylazine (2.25 mg/kg, 20 mg/ml, Sedazine, AST Farma, Oudewater, The Netherlands), Zoletil 100 (6 mg/kg, 100 mg/ml, Virbac, Barneveld, The Netherlands), injected intramuscularly. Then pentobarbital 50 mg/ml (10 to 15 mg/kg/h, Faculty Pharmacy Utrecht, The Netherlands) was injected and the pig was intubated. Oxygen (25–30% v/v) and nitrogen (75–80% v/v) were provided through ventilation to keep blood gas values within physiological ranges. To maintain anaesthesia, isoflurane (1–2.5% v/v) was administered through ventilation.

250 mg of acetylsalicylic acid (Aspegic, Sanofi-Aventis) and 10,000 units of heparin (Heparin Leo, Leo Pharma, Amsterdam, The Netherlands) were administered through a sheath deployed in the carotid artery of the animal, with an extra dose of 5000 units of heparin every hour.

Through the same sheath, an 8 Fr JL3.5 guiding catheter (Boston Scientific, Marlborough, Massachusetts, USA) was inserted into the ostium of the LAD coronary artery. To induce vasodilation, isosorbide mononitrate (0.04 mg/kg, 1 mg/ml) was injected through the guiding catheter.

During the imaging experiment, the stents implanted in the LAD arteries of both animals, were imaged using both the commercial OCT system and the Heartbeat OCT system. The imaging catheter of the commercial OCT system (Dragonfly™ Duo, Abbott) was inserted over a 0.014″ 190 cm guidewire (HI-TORQUE PILOT 50, Abbott), while the Heartbeat OCT catheter was placed with a delivery catheter (GuideLiner V3 catheter, Vascular Solutions, Minneapolis, Minnesota, USA), as it is not guidewire-compatible. After the experiment, the animals were sacrificed and the coronary arteries were excised from the heart and frozen for further analysis.

### Micro CT imaging

In the frozen state the explanted vessels were imaged with an X-ray micro-computed tomography (μCT) scanner (Quantum FX, Perkin Elmer, Waltham, Massachusetts, USA), to provide a reference length measurement for the OCT images. The voxel size was set at 80 µm.

### Stent length comparison

The length of the deployed stent was measured in every OCT pullback, with the same modalities of the in vitro experiment. Using the proprietary software provided with the micro-CT scanner, the length of the frozen stents was measured as well and used as a reference. These lengths were then compared.

## Results

### Comparison of clinical OCT-measured lengths to nominal lengths

**Fig. 5:**
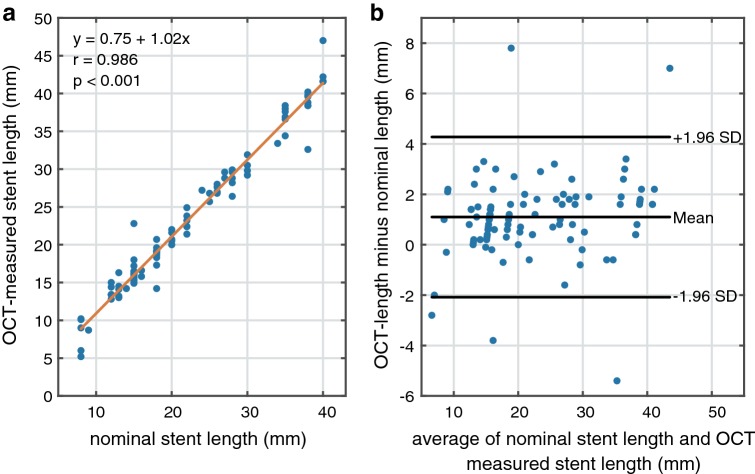
Nominal vs OCT-measured stent lengths in clinical data. **a** Regression analysis, **b** Bland–Altman plot

Regression analysis and Bland–Altman plots of the OCT-measured and nominal stents lengths are reported in Fig. 5. The nominal stent length ranged from 8 to 40 mm, average 21.7 ± 9.3 mm. The OCT-measured lengths varied from 5.2 to 47 mm, average 22.8 ± 9.6 mm. The average difference between the OCT-measured lengths and the nominal values was 1.1 ± 1.6 mm (r = 0.986, p < 0.001). The absolute maximum overestimated longitudinal measurement was 7.6 mm, the maximum underestimated measurement was 5.5 mm. Larger errors were observed in the RCA (1.6 ± 2.4 mm) compared to the LAD (1.0 ± 1.3 mm) and LCX (0.9 ± 1.2 mm). There was no difference in stent length measurement accuracy between 18 mm/s (N = 40) and 36 mm/s (N = 61) pullback speed.

### Cardiac motion simulation experiments

**Fig. 6:**
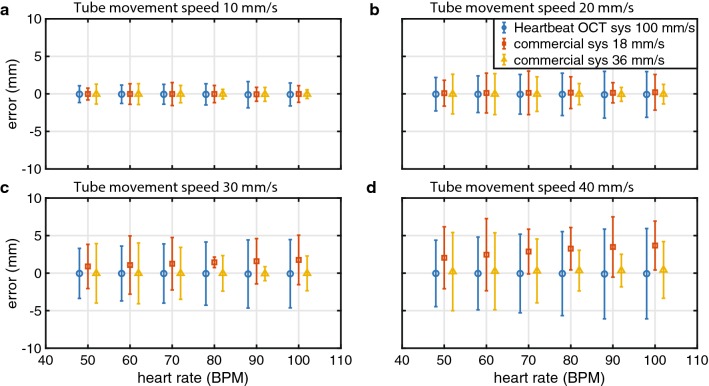
Numerical simulation of OCT-measured stent lengths, error ± standard deviation vs heart rate, for different tissue motion speeds. Nominal stent length 24 mm

Numerical simulation of OCT-measured stent lengths showed differences comparable to the clinically observed deviations from nominal length (Fig. 6). For low tissue velocities, the mean error vanished for all stent lengths and heart rates and the standard deviation of the difference between observed and nominal lengths was approximately 2 mm. The spread in the data increases with tissue velocity, and is slightly larger for the 36 mm/s pullback speed compared to 18 mm/s. The measured stent lengths are the most accurate with 100 mm/s pullback speed, even without synchronisation.

In vitro, the length of the deployed stent measured by OCT without motion was 25.3 ± 0.2 mm, in agreement with the calliper measurement of 25.35 mm. As in the numerical simulation, the relative movement between the stent and the imaging catheter introduces a measurement error, that increases in average and standard deviation at higher tissue velocity (Table 1). Synchronized Heartbeat OCT pullbacks showed a lower dispersion of the measurements. The acquisitions were triggered to coincide with the motion phase mimicking diastole, with minimal tissue motion. Thanks to the higher pullback speed, the Heartbeat OCT, for a range of pullback start delays, acquires the entire stent before the subsequent movement cycle starts, returning a measured length equal to the nominal value (Fig. 2b). Even with short cycles (100 BPM) when the zero-motion period is too short to image the entire stent in stationary conditions, the standard deviation of the measures is low due to the synchronisation.

**Table 1:** In vitro experiment results, reported as average measurement error ± standard deviation

	Tube speed = 10 mm/s 60 cycles/minute	Tube speed = 40 mm/s 60 cycles/minute
Commercial OCT pullback speed 18 mm/s	− 0.1 ± 1.2 mm	1.5 ± 4.5 mm
Commercial OCT pullback speed 36 mm/s	0.3 ± 1.3 mm	− 0.5 ± 4.9 mm
Heartbeat OCT pullback speed 100 mm/s	− 0.30 ± 0.03 mm	− 0.1 ± 0.1 mm

### In vivo imaging of stents

In two experiments, the stents implanted in the LAD of two swine were imaged. In each pig, one pullback with the commercial system and one with the Heartbeat OCT system were performed. In the first pig, the triggering of the Heartbeat OCT was synchronized on the animal ECG, and started 0.3 s after the R-peak; in the second the acquisition was asynchronous.

**Fig. 7:**
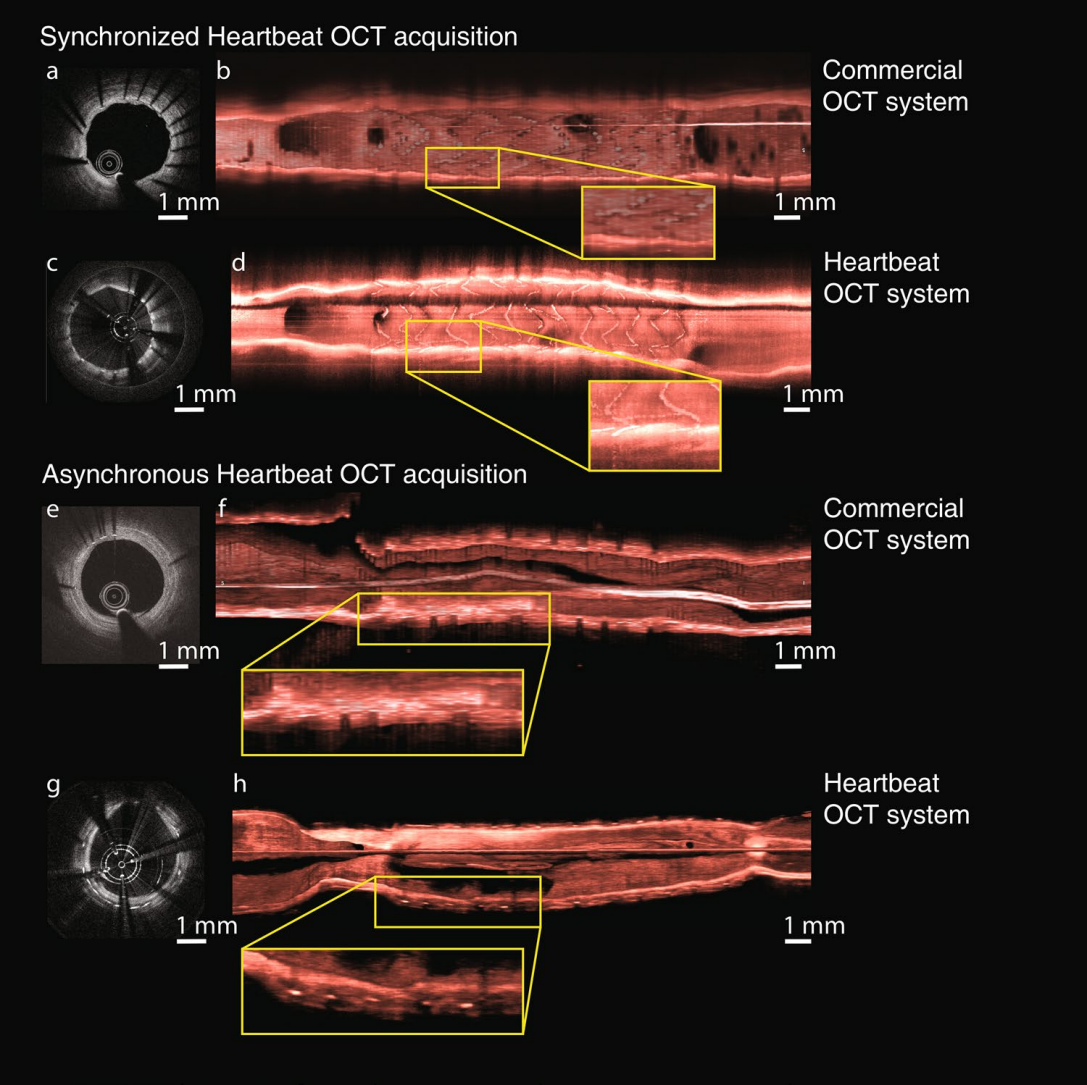
Cross-section images and 3D reconstructions of stents implanted in the LAD of two atherosclerotic swine. Top: OCT start synchronized to the ECG of the animal; bottom: asynchronous pullback

The delay used for the synchronized pullback was set heuristically based on motion patterns analysed from the angiographic images. The heart rate of the animals was 54 to 65 BPM in the first pig and 60 to 74 BPM in the second pig. One of the two data sets acquired with the Heartbeat OCT presented an incomplete flushing artefact, all other had adequate flush. Three-dimensional renderings of the imaged vessels are shown in Fig. 7. While the cross-sectional images (Fig. 7c and 7g) are affected by shadows due to the motor wires in the current design of the catheter, the longitudinal and 3D renderings of the data show a wealth of detail compared to the commercial system. In particular, because of improved longitudinal sampling, individual stent struts are clearly resolved, embedded in low-signal neo-intimal tissue (Fig. 7h), which has been associated with low cellularity and proteoglycan-rich tissue [26].

The recorded length of the stents are reported in Table 2. Without timed acquisition, the length measurement error in this 13 mm stent was about 0.9 mm in both the conventional and Heartbeat OCT systems. The stent length as measured in the synchronized Heartbeat OCT pullback was longer by 0.2 mm compared to the length measured in the μCT data, while the commercial system overestimated it by 1.2 mm.

**Table 2:** In vivo stent lengths, obtained with a µCT scanner, a commercial OCT system and the Heartbeat OCT system

Nominal (mm)	Micro-CT (mm)	Commercial OCT (mm)	Deviation	Heartbeat OCT	Asynchronous high-speed OCT	Deviation
13	12.80	14.0	1.2	13.0		0.2
13	12.32	11.4	− 0.9		11.5	− 0.8

## Discussion

In this study, we investigated the impact of cardiac motion on longitudinal measurements in OCT pullbacks. We found errors of typically 5.0%, highest in RCA (7.4%), lowest in LCX (4.1%), with outliers up to > 5 mm, in clinical data, independent of pullback speed. Similar errors could be produced by cardiac motion simulation and OCT imaging with both a commercial system and a custom-made OCT machine that performs pullbacks at high speed (Heartbeat OCT). Analysis of in vitro simulations, corroborated by illustrative in vivo measurements, demonstrated that high-speed (100 mm/s) pullbacks combined with synchronized acquisition can perform accurate length measurements in a moving vessel. The observed standard deviations of the (zero-mean) error in longitudinal measurements are smaller by an order of magnitude with the use of high-speed pullback in combination with ECG triggering, compared to asynchronous acquisition at conventional speeds. This may enable length measurements with an accuracy better than 100 μm in vivo, less than the frame pitch of today’s OCT systems.

In OCT-guided PCI, errors in length measurement may result in significant sizing and positioning issues. For instance, a stent in a side branch that is slightly too long or positioned too proximal may protrude in the lumen of the main branch, giving rise to increased risk of follow-up events [27] and potential complications when crossing wires in later procedures.

Our simulations confirm that the longitudinal measurement error increases with greater relative motion between the vessel and the catheter. This motion may be highly variable in practice, depending on the heart rate, cardiac phase, actual tissue displacement during systole, but also on the selectivity of the guiding catheter used to deliver the OCT catheter. A well-seated guiding catheter will move with the ostium and thus eliminate much of the relative motion between vessel and imaging catheter.

### Comparison of OCT-measured lengths to nominal lengths

We assessed the impact of motion artefacts in clinical OCT imaging in coronary arteries by measuring the length of implanted stents on OCT pullbacks and comparing those to the nominal length. In 2013, Liu et al. [19] conducted a similar analysis, with a comparable dataset. They found a smaller average difference of 0.15 mm, with a similar standard deviation of 0.68 mm. We attribute this small discrepancy to a different definition of stent edge: the first/last frame containing any number of stent struts in the present study, versus a requirement of at least 180° of continuous arc with visible struts in [19].

We performed a systematic study of cardiac motion and longitudinal measurement error using both a numerical model of catheter motion relative to a stented vessel and a physical bench-top implementation of this model. Both simulations showed the same trends: unbiased errors and increasing dispersion with heart rate and tissue motion. Inspection of the error distributions in both simulations showed that these are far from normal, in fact frequently bimodal. Thus, descriptive statistics in terms of the mean and standard deviation provide only a partial representation of the actual errors that may occur. The absolute errors measured in the bench top system were smaller by a factor of approximately two than the numerical simulations, and are closer to the clinically observed length deviations. We have no clear explanation for this difference.

The observations we made in the small number of in vivo pullbacks confirm that it is possible to make accurate stent length measurements during the diastolic phase of the cardiac cycle, and that asynchronous or slower acquisition degrade the reliability of the measurement. The small number of animals in this study precludes a quantitative analysis of in vivo imaging performance.

### In vivo imaging of stents

The data in Fig. 7 are the first images of stents in vivo acquired with the Heartbeat OCT system, showing neo-intima formation in this porcine model of coronary atherosclerosis in one animal, while in another there was minimal coverage of the struts at 28 days after implantation. The images show a high level of detail.

The Heartbeat OCT catheter is a prototype which cannot be directly applied for clinical imaging. Several improvements, related to catheter size, deliverability and the presence of shadows in the image, will be implemented in future versions of the device.

ECG triggering and a higher acquisition speed improve the longitudinal fidelity of OCT data sets, overcoming limitations such as cardiac motion artefacts and undersampling that affect length measurements and volumetric renderings in conventional implementations of the technology.

## References

[1] Mintz GS (1996). Limitations of angiography in the assessment of plaque distribution in coronary artery disease: a systematic study of target lesion eccentricity in 1446 lesions. Circulation.

[2] Marrocco CJ (2012). Intravascular ultrasound. Semin Vasc Surg.

[3] Huang D (1991). Optical coherence tomography. Science.

[4] Tearney GJ (2012). Consensus standards for acquisition, measurement, and reporting of intravascular optical coherence tomography studies: a report from the International Working Group for Intravascular Optical Coherence Tomography Standardization and Validation. J Am Coll Cardiol.

[5] Tearney GJ (1996). Scanning single-mode fiber optic catheter-endoscope for optical coherence tomography. Opt Lett.

[6] Yun SH (2006). Comprehensive volumetric optical microscopy in vivo. Nat Med.

[7] Jang IK, Tearney G, Bouma B (2001). Visualization of tissue prolapse between coronary stent struts by optical coherence tomography: comparison with intravascular ultrasound. Circulation.

[8] di Mario C, Koskinas KC, Räber L (2018). Clinical benefit of IVUS guidance for coronary stenting: the ULTIMATE step toward definitive evidence?. J Am Coll Cardiol.

[9] Hong SJ (2015). Effect of intravascular ultrasound-guided vs angiography-guided everolimus-eluting stent implantation: the IVUS-XPL randomized clinical trial. JAMA.

[10] Zhang J (2018). Intravascular ultrasound versus angiography-guided drug-eluting stent implantation: the ULTIMATE trial. J Am Coll Cardiol.

[11] Jones DA (2018). Angiography alone versus angiography plus optical coherence tomography to guide percutaneous coronary intervention: outcomes from the Pan-London PCI Cohort. JACC Cardiovasc Interv.

[12] Prati F (2012). Angiography alone versus angiography plus optical coherence tomography to guide decision-making during percutaneous coronary intervention: the Centro per la Lotta contro l'Infarto-Optimisation of Percutaneous Coronary Intervention (CLI-OPCI) study. EuroIntervention.

[13] Ali ZA (2016). Optical coherence tomography compared with intravascular ultrasound and with angiography to guide coronary stent implantation (ILUMIEN III: OPTIMIZE PCI): a randomised controlled trial. Lancet.

[14] Meneveau N (2016). Optical coherence tomography to optimize results of percutaneous coronary intervention in patients with non-ST-Elevation acute coronary syndrome: results of the Multicenter, Randomized DOCTORS Study (Does Optical Coherence Tomography Optimize Results of Stenting). Circulation.

[15] Andreasen LN, Balleby IR, Holm NR (2015). Uncertain detection of nonuniform scaffold expansion patterns using optical coherence tomography. JACC Cardiovasc Interv.

[16] Wang T (2015). Heartbeat OCT: in vivo intravascular megahertz-optical coherence tomography. Biomed Opt Express.

[17] Wang T, van Soest G, van der Steen AFW (2015). A micromotor catheter for intravascular optical coherence tomography. Engineering.

[18] Kim TS (2016). Single cardiac cycle three-dimensional intracoronary optical coherence tomography. Biomed Opt Express.

[19] Liu Y (2014). Comparison of longitudinal geometric measurement in human coronary arteries between frequency-domain optical coherence tomography and intravascular ultrasound. Int J Cardiovasc Imaging.

[20] Wang T (2013). Intravascular optical coherence tomography imaging at 3200 frames per second. Opt Lett.

[21] Huber R, Wojtkowski M, Fujimoto JG (2006). Fourier Domain Mode Locking (FDML): a new laser operating regime and applications for optical coherence tomography. Opt Express.

[22] Wang T, van Soest G, van der Steen AFW (2015). A micromotor catheter for intravascular optical coherence tomography. Engineering.

[23] Dandel M (2009). Strain and strain rate imaging by echocardiography—basic concepts and clinical applicability. Curr Cardiol Rev.

[24] Garber JC (2011). Guide for the care and use of laboratory animals.

[25] Thim T (2010). Familial hypercholesterolaemic downsized pig with human-like coronary atherosclerosis: a model for preclinical studies. EuroIntervention.

[26] Ughi GJ (2013). Automatic characterization of neointimal tissue by intravascular optical coherence tomography. J Biomed Opt.

[27] Prati F (2015). Clinical impact of OCT findings during PCI: the CLI-OPCI II study. JACC Cardiovasc Imaging.

